# Active Learning Approach for Guiding Site-of-Metabolism Measurement and Annotation

**DOI:** 10.1021/acs.jcim.3c01588

**Published:** 2024-01-03

**Authors:** Ya Chen, Thomas Seidel, Roxane Axel Jacob, Steffen Hirte, Angelica Mazzolari, Alessandro Pedretti, Giulio Vistoli, Thierry Langer, Filip Miljković, Johannes Kirchmair

**Affiliations:** † Department of Pharmaceutical Sciences, Division of Pharmaceutical Chemistry, Faculty of Life Sciences, University of Vienna, Josef-Holaubek-Platz 2, 1090 Vienna, Austria; ‡ Christian Doppler Laboratory for Molecular Informatics in the Biosciences, Department for Pharmaceutical Sciences, University of Vienna, 1090 Vienna, Austria; § Vienna Doctoral School of Pharmaceutical, Nutritional and Sport Sciences (PhaNuSpo), University of Vienna, 1090 Vienna, Austria; ∥ Dipartimento di Scienze Farmaceutiche, Università degli Studi di Milano, I-20133 Milano, Italy; ⊥ Medicinal Chemistry, Research and Early Development, Cardiovascular, Renal and Metabolism (CVRM), BioPharmaceuticals R&D, AstraZeneca, Pepparedsleden 1, SE-43183 Gothenburg, Sweden

## Abstract

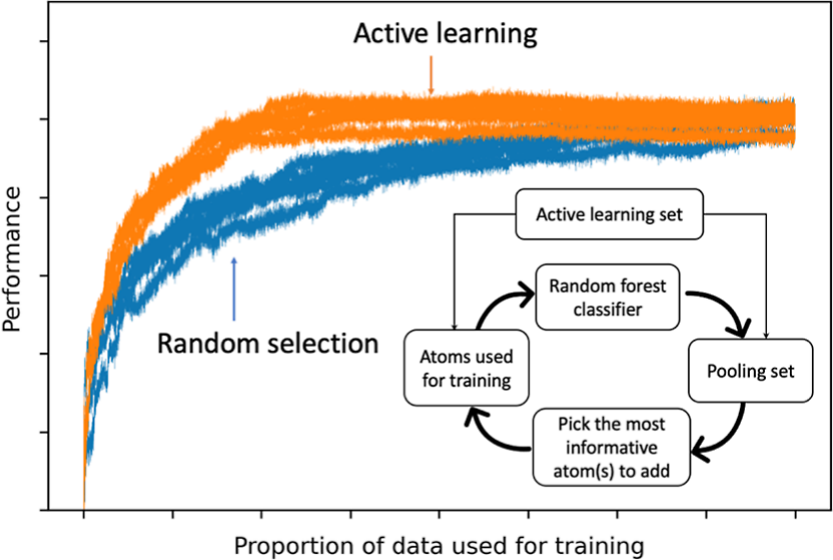

The ability to determine and predict metabolically labile atom positions in a molecule (also called “sites of metabolism” or “SoMs”) is of high interest to the design and optimization of bioactive compounds, such as drugs, agrochemicals, and cosmetics. In recent years, several in silico models for SoM prediction have become available, many of which include a machine-learning component. The bottleneck in advancing these approaches is the coverage of distinct atom environments and rare and complex biotransformation events with high-quality experimental data. Pharmaceutical companies typically have measured metabolism data available for several hundred to several thousand compounds. However, even for metabolism experts, interpreting these data and assigning SoMs are challenging and time-consuming. Therefore, a significant proportion of the potential of the existing metabolism data, particularly in machine learning, remains dormant. Here, we report on the development and validation of an active learning approach that identifies the most informative atoms across molecular data sets for SoM annotation. The active learning approach, built on a highly efficient reimplementation of SoM predictor FAME 3, enables experts to prioritize their SoM experimental measurements and annotation efforts on the most rewarding atom environments. We show that this active learning approach yields competitive SoM predictors while requiring the annotation of only 20% of the atom positions required by FAME 3. The source code of the approach presented in this work is publicly available.

## Introduction

Xenobiotic metabolism can determine the efficacy and safety of bioactive small organic compounds, such as drugs, agrochemicals, and cosmetics. Today, powerful experimental approaches for determining the biotransformation of small organic molecules are in place but remain resource-intensive and time-consuming. Therefore, in silico models for the prediction of xenobiotic metabolism are of great interest to researchers involved in the design and optimization of bioactive compounds.^1^ In particular, predictors of sites of metabolism (SoMs), i.e., the atom positions in a molecule where metabolic reactions are initiated (“metabolic hotspots”), continue to draw significant attention. Once the (likely) SoMs in a molecule are identified, medicinal chemists can often devise strategies for optimizing the metabolic properties while maintaining the compound’s bioactivity on the biomacromolecular target. Likewise, some metabolite structure predictors (including GLORYx,^2^ Meteor,^3−5^ and XenoNet^6,7^) use predicted SoMs to filter and rank predicted metabolites.

Several SoM predictors are available today, most of which involve machine learning components: ADMET Predictor Metabolism module,^8^ FAME,^9^ MetaSpot,^10^ MetaSite,^11^ the P450 SoM Predictor of the Schrödinger platform,^12^ SMARTCyp,^13^ SOMP,^14^ the StarDrop P450 Metabolism Prediction module,^15^ and the XenoSite platform.^16,17^

On holdout data (i.e., compounds with annotated SoMs), the leading SoM predictors typically rank at least one experimentally observed SoM among the two top-ranked atom positions (“top-2 metric”) for at least 80% of the test compounds. Note that the data used for model testing usually represent a similar chemical space to the training data. Hence, the performance of the models on data representing innovative chemical spaces will likely be overestimated by these tests. However, recent studies^9,18^ show that the applicability domain of SoM predictors is broader than that of many molecular property predictors. The broad applicability of SoM predictors is related to the fact that metabolic liability is a function of the proximate atom environment and these local environments are, to some extent, redundant across chemical spaces.

Most noncommercial SoM predictors are trained on the same set of 680 drugs and drug-like compounds with experimentally determined, expert-curated SoMs: the Zaretzki data set.^16^ Although of enormous value to the scientific community, the Zaretzki data set covers only cytochrome P450 (CYP)-mediated metabolism. While CYPs certainly are the most relevant xenobiotic-metabolizing enzymes, there are many other phase 1 enzymes (e.g., reductases and hydrolases) and also phase 2 enzymes (mainly transferases) that are of high relevance to small-molecule research.^19^ One of the few SoM predictors with comprehensive coverage of phase 1 and phase 2 metabolism is FAME 3, which was developed by some of us. FAME 3 is trained on 1733 parent compounds with experimentally determined, expert-curated SoMs for phase 1 and phase 2 metabolic enzymes (i.e., the MetaQSAR database^20^).

**Figure 1:**
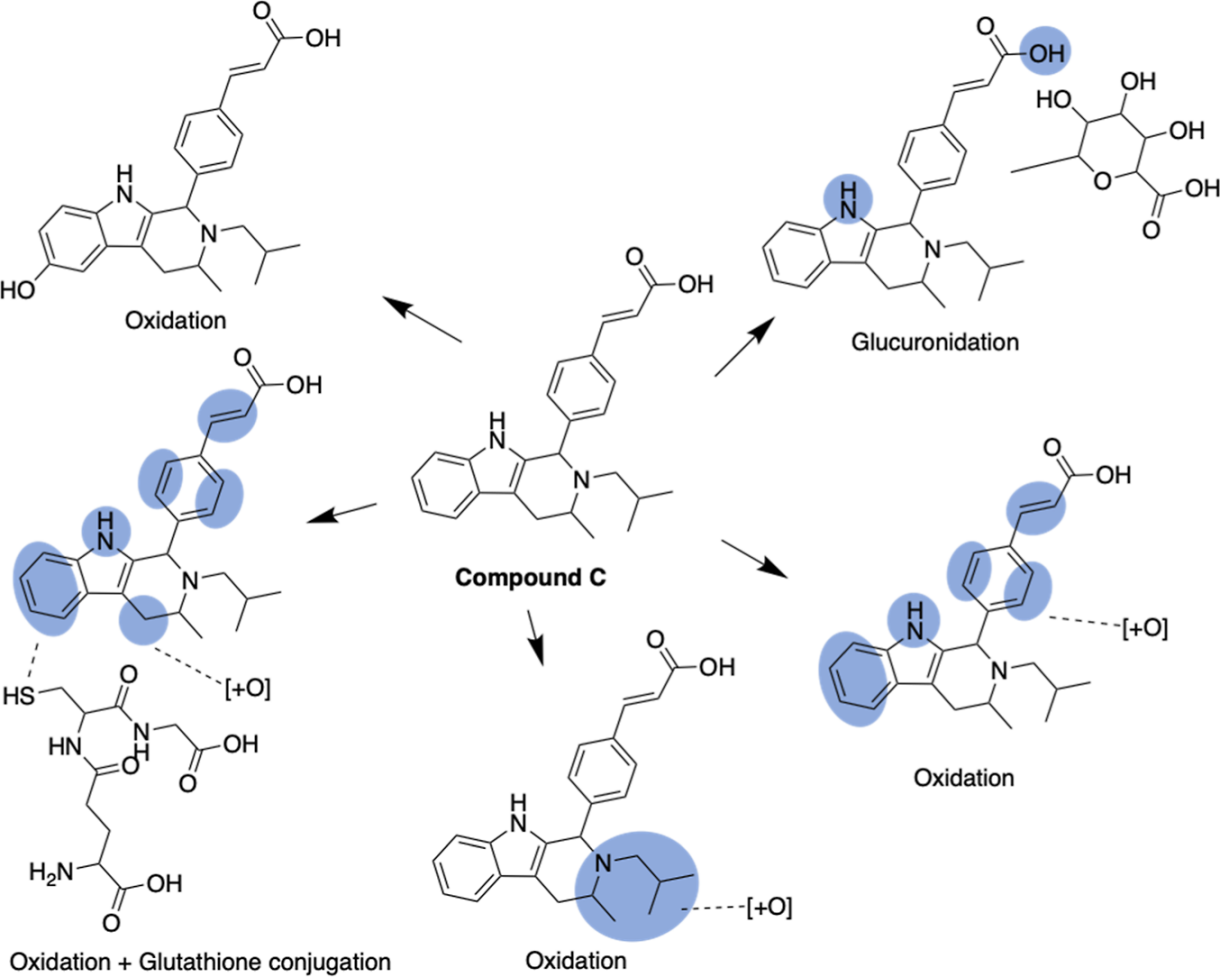
Visualization of the results of a representative mass spectrometry study of a drug-like compound, published in ref (25). The blue circles indicate areas in the molecules where metabolic reactions are experimentally observed. Several of these areas span across two or more atoms, reflecting the uncertainty in the measured data about the exact location of SoMs.

Significant advances in the accuracy and applicability of SoM predictors will depend on the availability of additional measured metabolism data on distinct atom environments, particularly those involved in rare complex biotransformation events. However, the costs associated with generating metabolism data are substantial. Typically, the identification of SoMs involves liquid chromatography–mass spectrometry (LC–MS) experiments and experts’ diligent and time-consuming work to interpret the data and deal with uncertainty about the exact atom position of some biotransformations (Figure 1). Therefore, it is unlikely that the rate at which measured data become available in the public domain will improve dramatically over the next few years.

Pharmaceutical companies, where most drug metabolism research is conducted today, typically have access to measured raw data on the metabolism of several hundred to several thousand (mostly) proprietary compounds. However, we are unaware of any research institution having tasked experts with systematically interpreting their measured raw data to annotate SoMs. In this context, a computational method that cherry-picks, across molecular data sets, the most informative atoms for experts to annotate (or measure) could be a game-changer for SoM predictor development. It could reduce the need for measuring and annotating the metabolic stability of the atoms of as many compounds as possible into the need for selectively measuring and annotating only the most informative atoms across sets of molecules.

One powerful approach for cherry-picking the most informative data samples in machine learning is active learning. During active learning, a machine learning model guides the acquisition of additional data for model training in an iterative process. More specifically, a machine learning model is trained on a small portion of the available data. Then, the model iteratively selects the most informative sample (or batch of samples) to acquire in preparation for the next cycle of model training. Recent successful applications of active learning strategies in cheminformatics include high-throughput docking,^21^ as well as the ligand-based prediction of physicochemical and biological properties.^22−24^

In this work, we show that active learning requires only 20% of the SoM/non-SoM labeled atoms used by classical approaches (in this case, FAME 3) to reach competitive performance. In other words, the active learning approach enables researchers to fully benefit from their raw metabolism data (i.e., 100% of their parent compounds with measured metabolism data) while requiring expert SoM annotations for only 20% of the atoms in their data set. The active learning approach also enables experimentalists to focus their experimental data acquisition on the most informative atom positions across a set of compounds.

## Methods

The active learning approach builds on FAME 3 and the observations made during its development and validation. The data processing workflow, atomic descriptor calculation, and machine learning procedure employed in FAME 3 were refined in preparation for active learning, as summarized in Table 1 and discussed in the following sections.

**Table 1:** Comparison of Key Technical Facts of the Models Presented in This Work and FAME 3

	FAME 3	this work
primary programming language	Java	Python
software libraries	CDK, scikit-learn	CDPKit, RDKit, scikit-learn
data	MetaQSAR database	MetaQSAR database
descriptors	15 atomic descriptors calculated with CDK	15 atomic descriptors calculated with CDPKit (Table S2), 14 thereof identical with those from CDK; stabilizationPlusCharge descriptor replaced with CDPKit’s inductive effect descriptor
bond path length of the atomic descriptors	5	1, 3, 5, 7^a^
machine learning algorithm	extremely randomized trees	random forest
number of estimators	250	250
class weight	balanced_subsample	balanced_subsample
decision threshold	0.40	0.30

a For the baseline model, a bond path length of 5 was used.

### Data Sets and Structure Processing

SoM data were extracted from the MetaQSAR database,^20,26^ composed of 2314 parent compounds with annotated SoMs. Any compound violating at least one of the following criteria was removed from the data set:

- Compound has at least one experimentally confirmed SoM annotated.
- Compound is composed exclusively of the following element types: C, N, S, O, H, F, Cl, Br, I, P, B, and Si.
- Compound has a molecular weight between 100 and 1000 Da.
- Compound can be successfully parsed with RDKit^27^

The molecular structures were standardized and salt components were removed with the ChEMBL Structure Pipeline.^28,29^ After the removal of any stereochemical information, duplicate molecular structures were identified and merged based on their InChI representations. As part of the deduplication process, SoM annotations were merged by using the GetSubstructMatches function of RDKit, taking topological symmetry into account. This procedure resulted in a processed data set of 1926 compounds (Table 2).

**Table 2:** Composition of the Data Sets Used in this Work and the FAME 3 Validation Study

	no. substrates	no. heavy atoms	no. SoMs	average no. SoMs per molecule	fraction of SoMs among heavy atoms
preprocessed data set (total)	1926	43 418	4976	2.58	0.11
training set (subset)	1505	33 994	3930	2.61	0.12
test set (subset)	421	9424	1046	2.48	0.11
FAME 3 P1 + P2 data set^a^ (total)	2167	49 045	6307	2.91	0.13
training set^a^ (subset)	1733	39 131	n/a	n/a	n/a
test set^a^ (subset)	434	9914	n/a	n/a	n/a

a Values obtained from ref (9).

**Figure 2:**
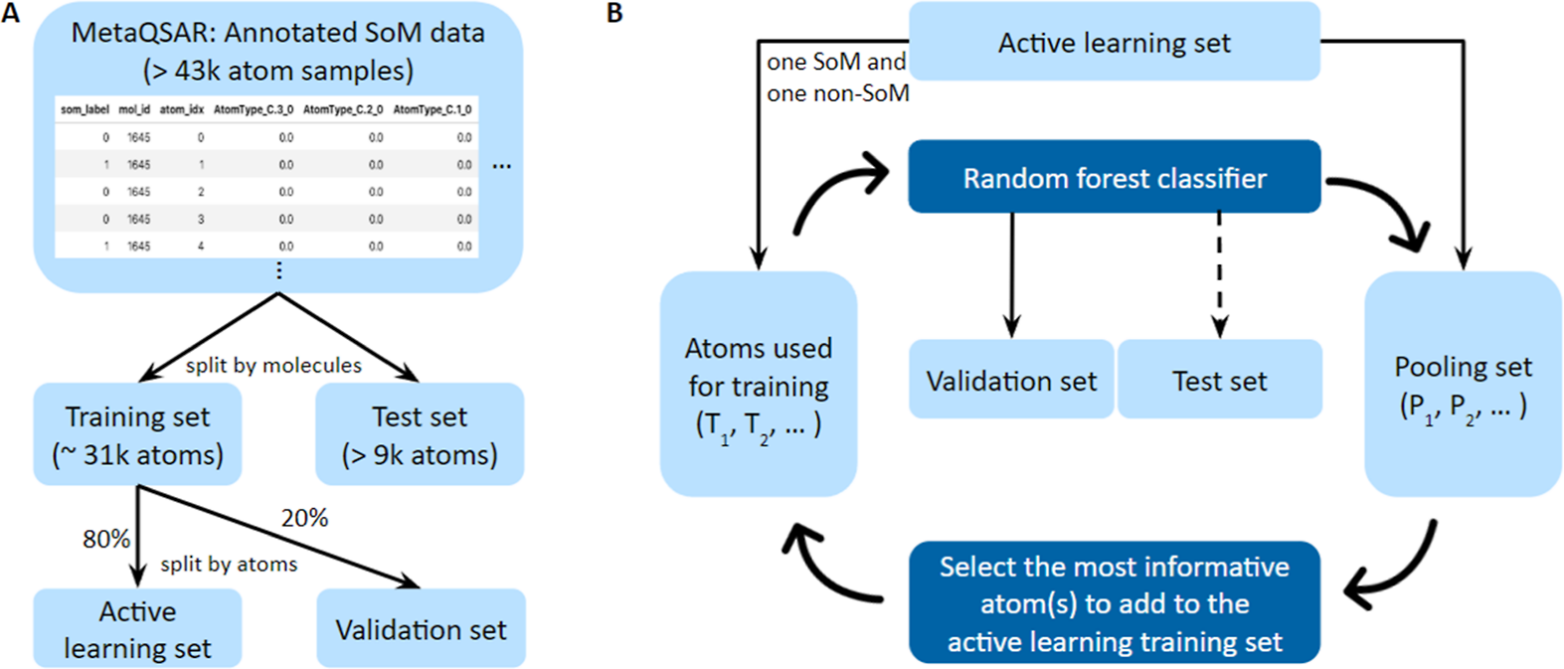
Overview of the (A) data sets and (B) active learning process employed in this work. (A) All compounds in the MetaQSAR database and annotated with SoM labels were split (by molecule) into a training set and a test set. These two subsets of the MetaQSAR database were used to train the baseline model and compare its performance with that of FAME 3. For active learning, the training set was further split (by atom) into an active learning set and a validation set. (B) During the iterative active learning process, the most informative *n* atoms (where *n* is greater than or equal to 1) are selectively added to the active learning, while all remaining ones serve as the data pool for later iterations of the selection process. The validation set was used to evaluate the performance of every generated model, whereas the test set was utilized to evaluate the performance of selected models only.

The processed data set was split into a training and a test set (Figure 2A). To support the comparability of this work with the previously published validation study on FAME 3,^9^ we maintained the identical split of the training and test data. However, because of a refined data merging procedure, which accurately detects any topologically equivalent molecules and combines the SoM annotations of topologically identical atoms within molecules without information loss, the numbers of compounds in the training and test data sets differ from those published for the FAME 3 validation study (Table 2).

### Descriptor Calculation

A set of 15 atomic descriptors was calculated with CDPKit^31^ (“CDPKit FAME descriptor set”). This set comprises one Sybyl atom type descriptor (discriminating 24 combinations of element types and hybridization states; Table S1) and 14 electronic and topological descriptors (Table S2). The descriptor set is similar to that used in FAME 3. However, in FAME 3, the descriptors are calculated with CDK^30^ instead of CDPKit, and CDK’s stabilizationPlusCharge descriptor is used instead of CDPKit’s inductive effect descriptor. In addition to this set of 15 atomic descriptors, the FAME fingerprint,^9^ which is a circular, atom-based binary fingerprint, was reimplemented in CDPKit and used in one instance of data set splitting (see the section “Active Learning”).

### Generation of a Baseline Model

A random forest classifier for SoM prediction was built with scikit-learn.^32^ For this classifier, the hyperparameters were adopted from FAME 3 (see Table 1), except for the decision threshold, which was reduced from 0.4 to 0.3 (the value of 0.4 originates from the work on FAME 2,^18^ which is based on a different SoM data set with a different class balance; meanwhile, we found that for MetaQSAR-derived models a value of 0.3 produces slightly better results). Furthermore, random forests were utilized instead of extremely randomized trees (FAME 3).

### Active Learning

Active learning was performed on the training data within a 5-fold cross-validation (CV) framework using the identical modeling algorithm (i.e., random forest), hyperparameters, and descriptors as we used for the baseline model (Figure 2). Two methods for generating the folds (which form the active learning and validation sets) based on atoms were explored: StratifiedKFold (as implemented in scikit-learn), which preserves the ratio of SoMs and non-SoMs in each fold, and clustering by atom similarity, which uses Butina clustering^33^ of atom environments (represented by FAME fingerprints with a bond path length of 5) to generate five folds between which no atom pair exceeds a Tanimoto similarity threshold of 0.80. This approach ensures that no highly similar atoms are present between two folds.

Before splitting the data set into the active learning set and the validation set, a deduplication routine was executed that merges atoms for which the values of all descriptors are identical. The deduplication resulted in a training set of 30 509 atoms. No class label conflicts were observed. All relevant duplicate instances represent topologically symmetric atoms.

Following related work on active learning,^22^ the initial training set for active learning, *T*_1_, is generated by the random pick of a single SoM and a single non-SoM from the active learning set. All of the remaining data points from the active learning set serve as the pooling set, *P*_1_, and every atom in the pooling set is assigned a prediction value calculated with the model. Next, the most informative batch of annotated atoms [*a*_1_; atom(s) selected based on the distance of the prediction value from the decision threshold, without regard for the label] and the atoms contained in *T*_1_ are joined to form the next training set, *T*_2_. Likewise, *P*_2_ is formed by removing *a*_1_ from *P*_1_. This step is followed by the next training cycle, which uses *T*_2_ as the training set and *P*_2_ as the atom pool to select the next most informative batch of atoms. The process is iterated until all atoms from the pooling set have been selected and used for training. During each iteration, the model’s performance is evaluated on the validation set. Selected models are also evaluated on holdout data (the test set).

### Model Performance Metrics

The Matthews correlation coefficient (MCC) served as the primary metric for model performance assessment and optimization. It is one of the most robust and informative measures for evaluating binary classifiers because it is a balanced measure considering the proportion of all classes in the confusion matrix. Note that the MCC ranges from −1.0 to +1.0, with a value of 1.0 indicating an excellent classification performance.

In addition, the area under the receiver operating characteristic curve (AUC), which, in this context, quantifies the ability of a model to correctly rank SoMs and non-SoMs (based on the probabilities reported by the binary classifier), and the top-2 success rate, which, in this context, quantifies the proportion of molecules for which at least one known SoM is listed among the two top-ranked atom positions in a molecule (ranking according to the predicted probabilities of an atom to be a SoM), were calculated. Furthermore, recall (quantifying the proportion of SoMs that are corrected predicted), precision (indicating the proportion of true SoMs among all predicted SoMs), and Jaccard score (i.e., the ratio of the number of correctly predicted SoMs to the number of SoMs and the number of wrongly predicted non-SoMs; the higher the Jaccard score, the higher the accuracy of the classifier) were also evaluated.

## Results and Discussion

### SoM Prediction Performance of the FAME 3 Reimplementation – Baseline Model

To confirm the proper working of the reimplementation of FAME 3 with CDPKit descriptors and to establish a baseline for the evaluation of the active learning approach, we run tests with training and test data that are as closely as possible related to the training and test sets used in the FAME 3 validation study.^9^ More specifically, we preserved the data set split, but because of a refined data processing procedure (which corrects minor insufficiencies of the previously employed approach for the detection of identical molecules and topologically symmetric atoms), the subsets are similar but not identical (see Methods for details). The generated model is equivalent to the FAME 3 model covering phase 1 and phase 2 metabolism (referred to in the original publication as “FAME 3 P1 + P2 model”).

As reported in Table 3, the prediction performance of the new implementation is comparable with that of FAME 3 for both 10-fold CV and the test set. The results indicate that on average for more than 80% of the annotated compounds (from the validation set and the test set), at least one known SoM is found among the two highest-ranked atom positions in a molecule.

**Table 3:** Comparison of the Prediction Performance of FAME 3 with Reimplementation with CDPKit

model	MCC		AUC		top-2 (%)		average prediction time [s per molecule]^a^
	CV	test set	CV	test set	CV	test set	
FAME 3 P1 + P2^b^	0.51	0.50	0.89	0.90	82	82	∼13.2
reimplementation with CDPKit	0.50	0.50	0.88	0.89	81	82	∼0.1

a Averaged computing time per molecule with a single thread on a Linux workstation equipped with an AMD Ryzen 9 7950X 16-core CPU and 128 GB of RAM.

b Values are taken from ref (9).

Importantly, the reimplementation of FAME 3 with CDPKit is much faster than the original implementation, with an average prediction time per molecule of approximately 0.1 s compared to 13.2 s (measured on an AMD Ryzen 9 7950X CPU with a single thread of execution).

### Active Learning

We performed active learning using the identical machine learning setup we employed for building the baseline model (Table 1). The performance of the active learning approach was assessed by a 5-fold CV and with holdout data (test set). Two different methods for generating the individual CV folds were explored, using atoms as instances: a stratified splitting method, which ensures identical ratios of SoMs and non-SoMs across the individual folds, and a clustering method, which ensures that the atom environments represented in the individual folds are dissimilar (see Methods for details). It is expected that the latter splitting method produces more challenging data sets. Note that molecule-based splitting strategies were also explored but yielded inferior models and were not further pursued (Figure S1).

### Model Performance Progression

**Figure 3:**
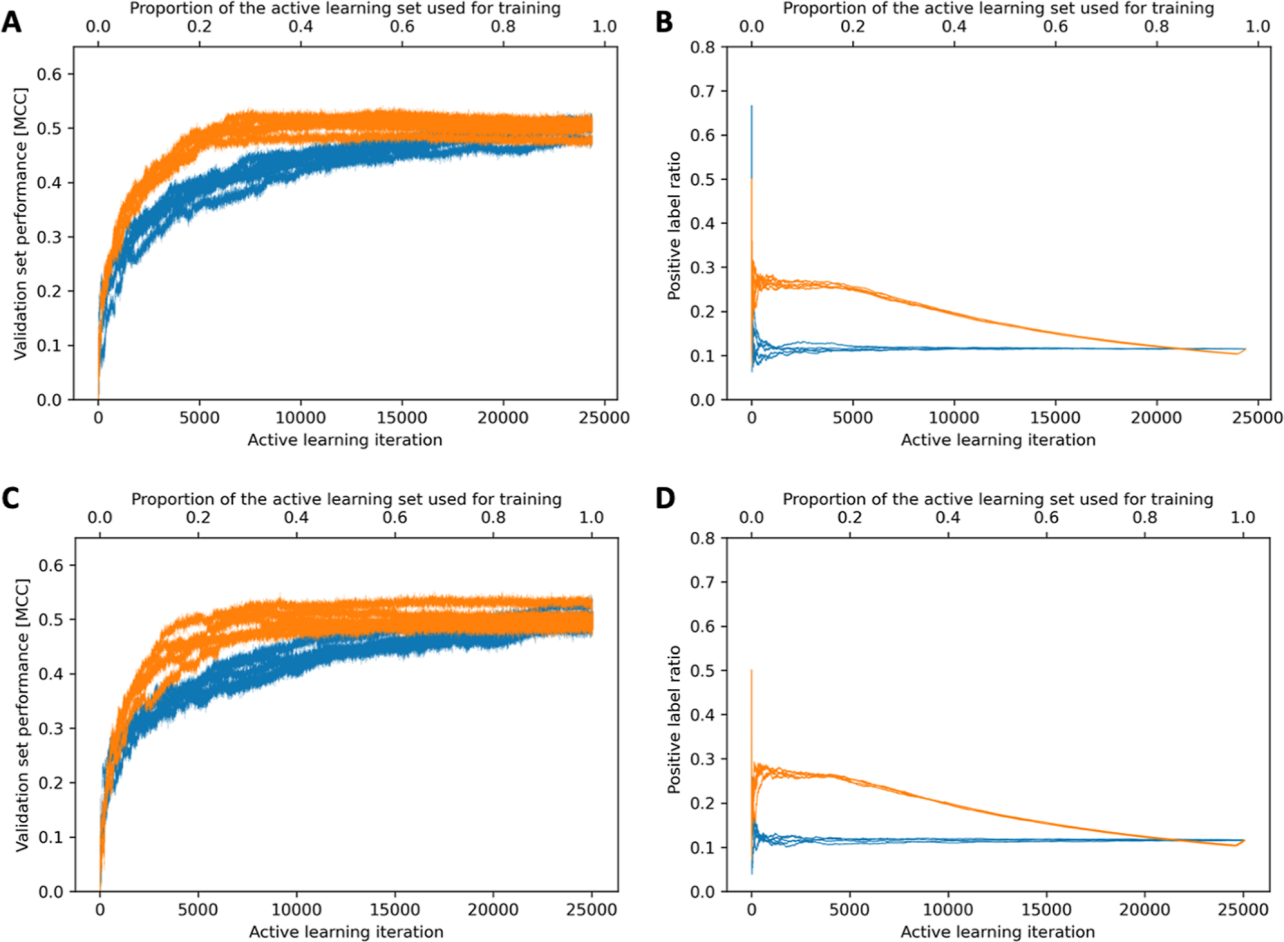
Performance progression of the active learning approach (orange) and the random selection approach (blue) as more data are used for model training. The five runs within the 5-fold CV framework are shown in each panel. (A,B) Performance progression when generating the data folds with StratifiedKFold; (C,D) performance progression when generating the data folds using clustering by atom similarity.

Active learning led to steep increases in model performance as more atoms were selected and used for model training (Figure 3). After approximately 6100 iterations (meaning the use of approximately 6100 atoms or 20%, of the data available for model building), the MCC reached 0.48 on the test set (Table 4), which is comparable to the MCC obtained by the baseline model (0.50). Also, the top-2 success rate, Jaccard score, and precision approached those of the baseline model when using just 20% of the training data (top-2 success rate 81 vs 82%; Jaccard score 0.37 vs 0.38; precision 0.55 vs 0.55; Tables 4 and S3). The AUC values and recall values approached equal levels for the test set (0.88 and 0.55, respectively) at a rather late stage of active learning, when approximately 40% of the data were used for training.

**Table 4:** Performance of Models as a Function of the Data Sampling Method and Training Set Size

data sampling method	% data used for model training^a^	data set^b^	MCC^c^		AUC^c^		top-2 (%)^c^		recall^c^		Jaccard score^c^	
			Mean	std	mean	std	mean	std	mean	std	mean	std
active learning	20/25	VS	0.49	0.01	0.84	0.00	n/a^d^	n/a^d^	0.53	0.02	0.37	0.01
active learning	20/25	TS	0.48	0.00	0.83	0.01	81	1	0.52	0.01	0.37	0.00
active learning	40/50	VS	0.51	0.02	0.88	0.01	n/a^d^	n/a^d^	0.55	0.03	0.39	0.02
active learning	40/50	TS	0.50	0.01	0.88	0.00	82	1	0.55	0.01	0.38	0.01
active learning	60/75	VS	0.50	0.01	0.89	0.00	n/a^d^	n/a^d^	0.56	0.03	0.39	0.01
active learning	60/75	TS	0.49	0.01	0.89	0.00	83	1	0.54	0.01	0.38	0.01
random selection	20/25	VS	0.40	0.02	0.85	0.01	n/a^d^	n/a^d^	0.43	0.03	0.30	0.02
random selection	20/25	TS	0.40	0.02	0.85	0.00	74	2	0.43	0.01	0.30	0.01
random selection	40/50	VS	0.46	0.02	0.88	0.00	n/a^d^	n/a^d^	0.49	0.04	0.35	0.02
random selection	40/50	TS	0.45	0.01	0.87	0.00	78	1	0.49	0.02	0.34	0.01
random selection	60/75	VS	0.48	0.01	0.89	0.00	n/a^d^	n/a^d^	0.52	0.02	0.37	0.01
random selection	60/75	TS	0.47	0.01	0.88	0.00	79	1	0.52	0.01	0.36	0.01
n/a^e^	80/100	VS	0.50	0.02	0.90	0.01	n/a^d^	n/a^d^	0.55	0.03	0.39	0.01
n/a^e^	80/100	TS	0.49	0.01	0.89	0.00	81	1	0.54	0.01	0.37	0.01
n/a^f^	100/n/a^f^	TS	0.50	n/a^f^	0.89	n/a^f^	82	n/a^f^	0.56	n/a^f^	0.38	n/a^f^

a of the training set for the baseline model/of the active learning set. For active learning, the training set used for generating the baseline model was further divided into an active learning set and a validation with a ratio of 80:20.

b VS: validation set; TS: test set.

c Performance averaged over five runs, each using a different fold as the validation set.

d Because splitting is performed on a per-atom basis and not on a per-molecule basis (meaning that for a given molecule, not all atoms may be represented in the validation set), top-2 success rates cannot be calculated.

e The complete active learning data set is used for model training.

f The complete training set for the baseline model; hence, the results are the performance of the baseline model on the test set.

The maximum MCC values recorded for the individual active learning runs were between 0.50 and 0.55 for the different validation sets (generated by the 5-fold split of the training set). For the test set, the MCC values were between 0.48 and 0.50 for the five repeats of active learning, meaning that the performance of the models generated with the active learning approach is comparable to that of the baseline model (MCC 0.50). The active learning approach did, under no circumstances, produce models superior in performance but generated competitive models with substantially less labeled data. For all approaches, performance was positively correlated with the size of the training or active learning set (Figure S2 illustrates this correlation for the active learning approach).

The standard deviations for the MCC maxima during the five repeats of active learning were just 0.01 when stratified random splitting was used to generate the five data folds and 0.02 when using the clustering approach. Because the stratified split method showed better stability during the active learning process, further discussion will focus on the results obtained with this data splitting method.

We compared the curve progression for the two sample selection strategies to confirm the added value of active learning over random sample selection (where a random atom instead of the most informative atom is added to the training set during each iteration). Figure 3 and Table 4 show that with smaller data sets active learning indeed produces better models: When using 6100 data points (i.e., 25% of the active learning data or 20% of the training set of the baseline model), the MCC values of the models generated with the two approaches were 0.49 and 0.40 for the validation sets, respectively, and 0.48 and 0.40 for the test set, respectively. The top-2 success rates, recall, Jaccard scores, and precision followed the same trend as the MCC values on the test set; only the performance improvement progression of the AUC values was comparable between the active learning and the random selection approach.

A further interesting curve progression to analyze is the positive label ratio. The positive label ratio quantifies the proportion of positive data (i.e., SoM data) among the samples selected for model training. SoM data sets have in common that the positive class is the minority class. In the case of the (processed) MetaQSAR data set, the fraction of SoMs is approximately 0.11 (Table 2). For the active learning approach, after an initial sharp peak, the positive label ratio quickly reaches a temporary equilibrium, just below the threshold value of 0.30. This equilibrium lasts for approximately 4000 iterations before gradually declining toward a value of 0.10. In contrast, the random selection approach maintains, again after an initial sharp peak, a positive label ratio of around 0.11. These observations show that the active learning approach’s data efficiency and performance advantage are not based solely on data balancing. When 20% of the atoms selected from active learning are utilized for model training, the positive label ratios are around 0.24. Still, on the validation sets, the predicted positive ratios were around 0.11, which aligns with the proportions of SoM atoms in the validation sets (Table S3).

### Robustness of the Approach with Respect to the Starting Points of Active Learning

**Figure 4:**
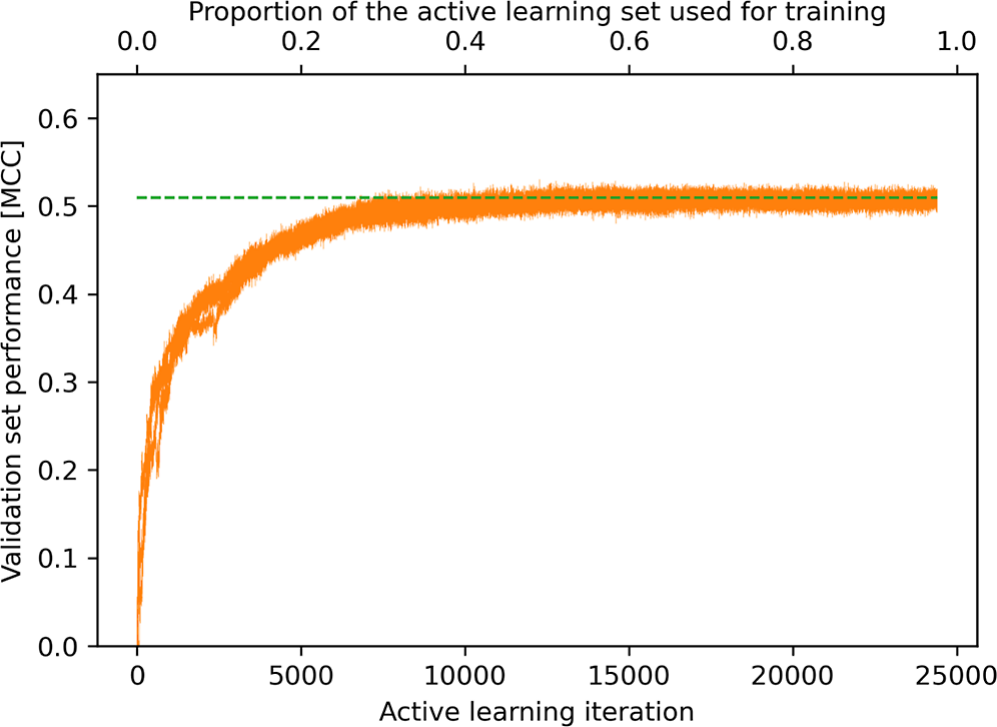
Variance in the performance progression of the active learning approach as different randomly selected pairs of atoms are used as a starting point for active learning. The graph shows five repeats of the complete active learning process as an example. The horizontal dashed line indicates the MCC obtained using the complete active learning set for model training.

To test the stability of the active learning approach with respect to the initial pair of atoms (one SoM and one non-SoM) selected for starting the iterative modeling process, we repeated the model building process five times (keeping fold 1 as the validation set for all five runs). As expected and shown in Figure 4 and Table 5, the differences between the individual runs with different initial atom pairs were marginal and became even smaller as further data were added to the training set. After approximately 6100 iterations already (representing 25% of the active learning data or 20% of the training set of the baseline model), the standard deviations of the MCC values were smaller than 0.01 in all cases.

**Table 5:** Stability of MCC Values when using Different Initial Training Samples

% data of the active learning set used in active learning	assessment interval^a^	MCC for repeat 1		MCC for repeat 2		MCC for repeat 3		MCC for repeat 4		MCC for repeat 5	
		mean	std	mean	std	mean	std	mean	std	mean	std
≥12.5	3050 to 24 406	0.49	0.02	0.50	0.02	0.49	0.02	0.50	0.02	0.50	0.02
≥25	6100 to 24 406	0.50	0.01	0.50	0.01	0.50	0.01	0.51	0.01	0.51	0.01
≥50	12 202 to 24 406	0.50	0.01	0.51	0.01	0.50	0.01	0.51	0.01	0.51	0.01
≥75	18 304 to 24 406	0.50	0.01	0.51	0.01	0.50	0.01	0.51	0.01	0.51	0.01

a Interval of iterations (from, to) for which the mean MCC and standard deviations are calculated.

**Figure 5:**
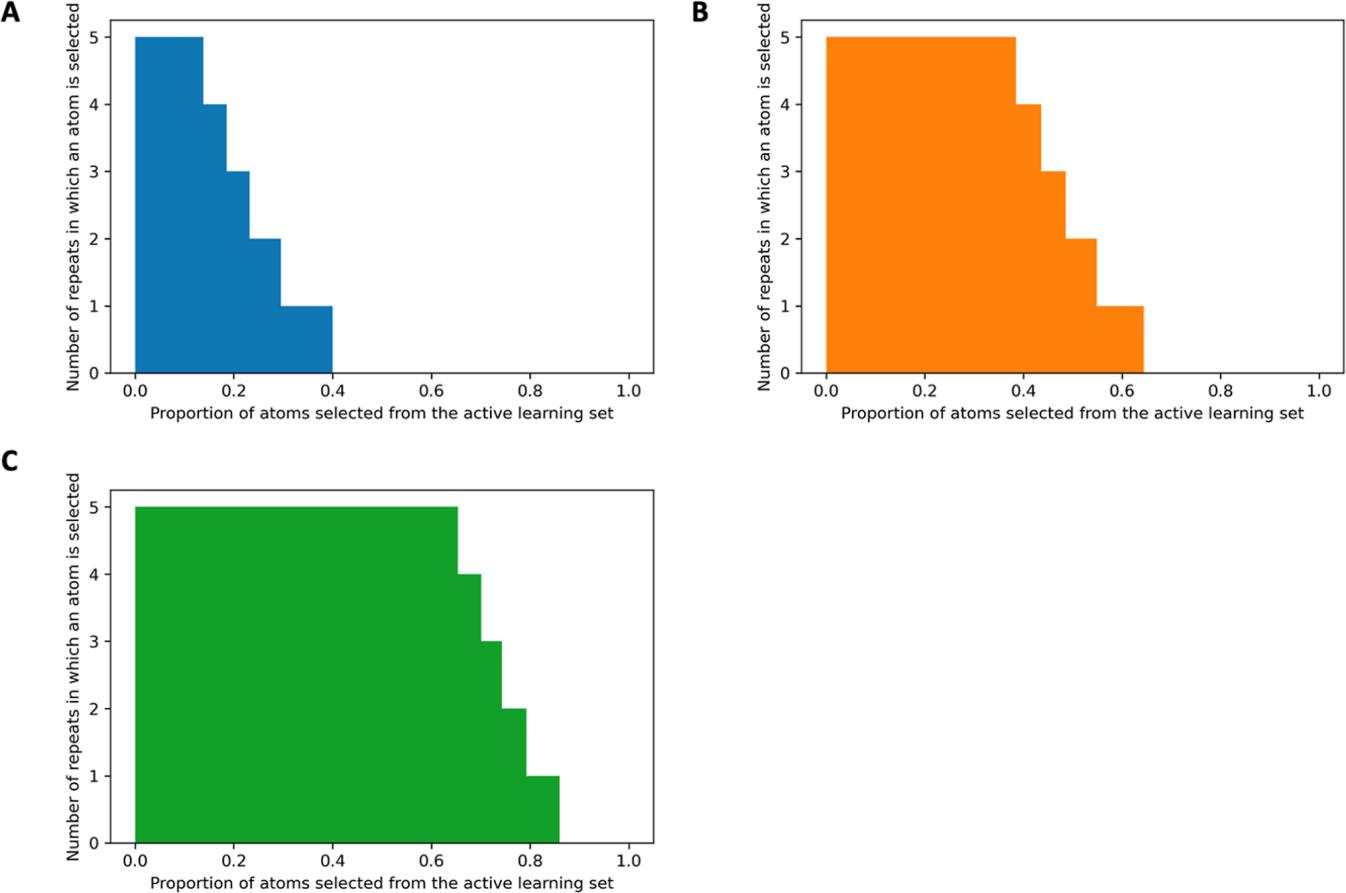
Number of times a specific atom was selected for model training during the five repeats of the complete active learning process at the time when (A) 25, (B) 50, and (C) 75% of the atoms in the active learning set were selected for model training.

The high stability of the active learning approach is also reflected by the fact that a substantial proportion of the atoms present in the active learning set were picked for training during each of the five repeats. For example, at the point when 25% of the atoms in the active learning set were selected for training, the number of atoms consistently selected during each of the (five) repeats of the experiment corresponded to 55% of the samples in the training data (Figure 5). The percentage of atoms consistently selected for training increased further with the progress of the active learning process. Based on these observations, we conclude that the active learning protocol is highly robust and yields consistent, good results largely independent of the starting conditions.

### Influence of Different Descriptor Bond Path Lengths on Model Performance

The performance of the active learning approach may be influenced by the size of the atom environments (defined by maximum bond path lengths) used to represent SoMs and non-SoMs with CDPKit FAME descriptors. Using the same experimental setup above, we explored how the active learning approach behaves using CDPKit FAME descriptors with bond path lengths of 1, 3, 5, and 7.

**Figure 6:**
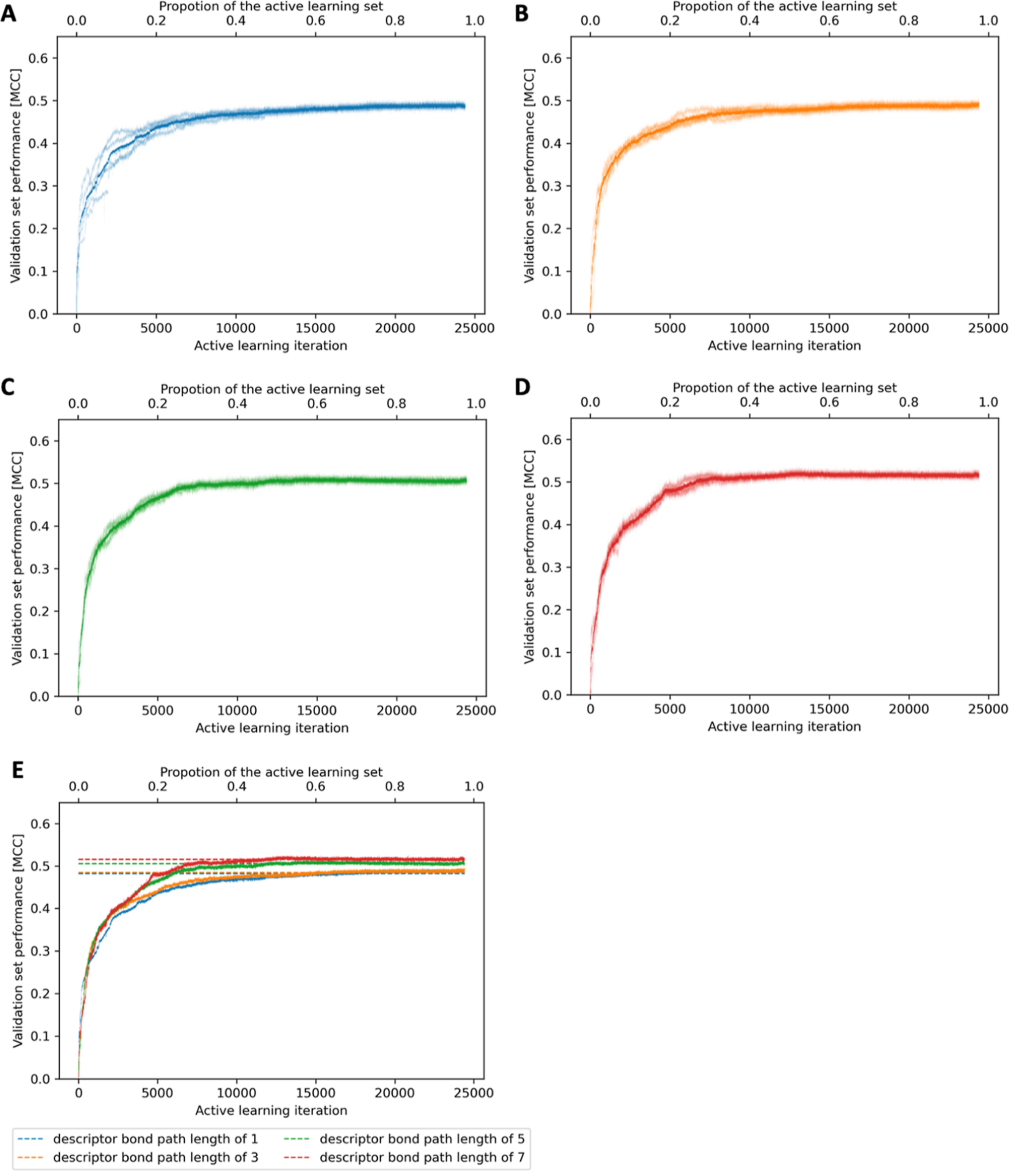
Performance of the active learning approach using descriptors with bond depths of (A) 1, (B) 3, (C) 5, and (D) 7 for five repeats of the complete active learning process. The averages of these five repeats with different bond path lengths are compared in (E). The horizontal dashed lines indicate the average MCC reached by the models after 10 000 iterations.

As shown in Figure 6, the active learning approach benefits slightly from using larger atom environments (meaning bond path lengths of 5 or 7). These resulted in steeper MCC curves with also a slightly higher MCC plateau (MCC of approximately 0.52 when using a bond path length of 7 vs 0.48 with a bond path length of 1 or 3). Regarding stability, a bond path length of 5 seems preferable over a bond path length of 7 (cp. Figures 6C,D), for which we conclude the optimum bond path length to be 5. This conclusion is consistent with the observations made for FAME 3.^9^

### Influence of Different Batch Sizes and Methods on Model Performance

**Figure 7:**
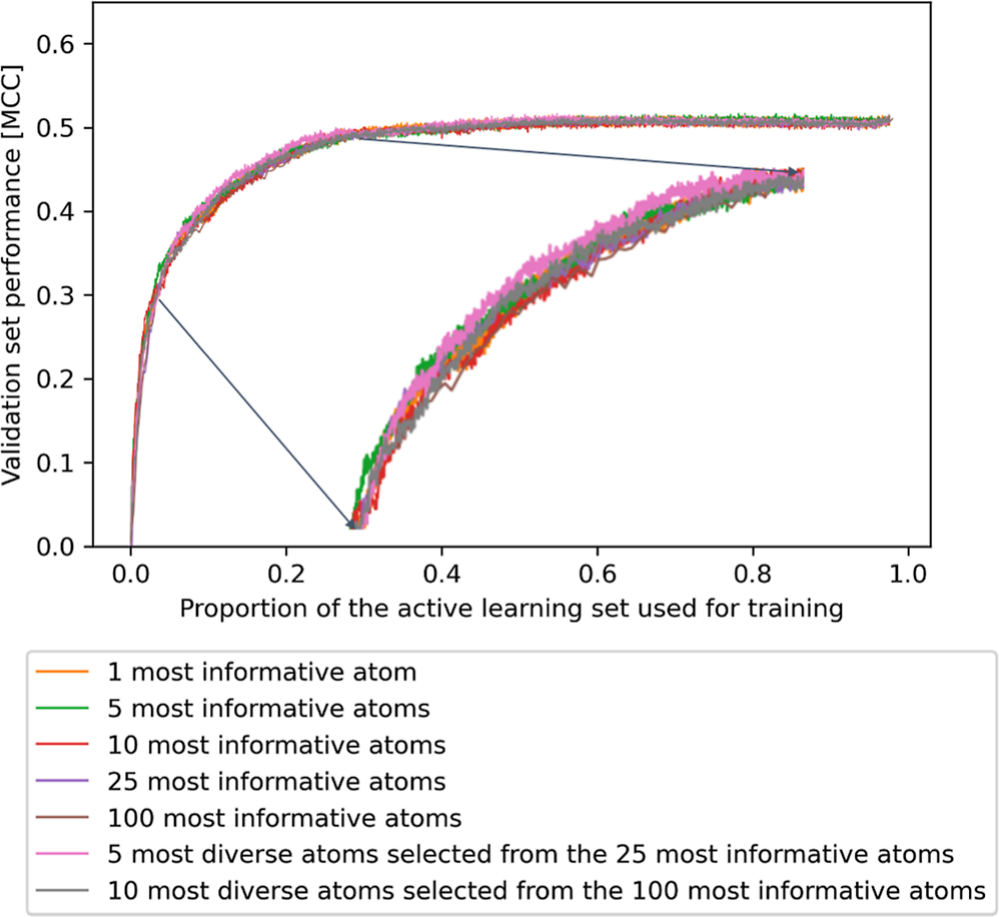
Impact of different batch sizes on active learning performance measured as MCC averaged over five repeats. Shown are runs with batches composed of the (i) 1, 5, 10, 25, or 100 most informative atom(s), (ii) 5 most diverse atoms selected from the 25 most informative atoms, and (iii) 10 most diverse atoms from the 100 most informative atoms. Because of the high stability of the repeats, only averages are reported. Note that the curves for runs with larger batch sizes are supported by fewer data points because the active learning process has fewer iterations.

On an AMD Ryzen 9 7950X CPU, one complete active learning run with the MetaQSAR data set takes approximately 1 day with eight threads of execution. To increase the computational efficiency of the approach, we investigated the use of larger batch sizes during active learning (the batch size used thus far is one atom). We explored two strategies to generate larger batches: mini-batch, which produces a batch from a defined number of most informative atoms and diverse mini-batch,^34^ which composes a batch from a defined number of diverse, most informative atoms (identified by *k*-means^35^ clustering). For mini-batch, we explored batch sizes of 5, 10, 25, and 100 atoms; for a diverse mini-batch, we explored batches generated from the 5 most diverse atoms selected from the 25 most informative atoms and from the 10 most diverse atoms selected from the 100 most informative atoms. Following the identical active learning setup as in the previous experiments, batch sizes of up to 100 showed similar trends for the performance progressions (Figure 7). The diverse mini-batch sampling did not offer an advantage (e.g., a steeper performance increase) over the standard mini-batch approach. From this, we conclude that adding small batches of atoms instead of single atoms is well-tolerated, leading to a substantial speedup of the active learning process.

## Conclusions

Methods and models for SoM prediction have come a long way. The performance and applicability of the leading SoM predictors surpass those of many predictors of other molecular properties. Furthermore, substantial progress in the field will depend on additional high-quality data on small-molecule metabolism. Given the considerable demands in experimental resources and expertise, a theoretical approach enabling researchers to focus their resources for measurement and annotation on the most informative atom environments is urgently needed.

We have devised an active learning approach that reaches competitive performance (MCC of 0.48 on holdout data) while using 80% less data than FAME 3 for model training. The active learning approach is robust with regard to initialization and model parameters. Its efficiency can be further increased by adding small batches of annotated atoms rather than a single atom during each iteration of active learning.

Researchers with access to raw metabolism data on small molecules can use the active learning approach to prioritize SoM annotation of cherry-picked atoms, whereas experts with access to HPLC-MS will benefit from the approach’s capacity to cherry-pick the most informative molecules and atoms for experimental testing. The active learning approach can transform the task of measuring and annotating the atoms of as many molecules as possible into a task involving the investigation of only the most informative and, hence, most rewarding atom positions.

We hope that our active learning approach, for which we release the complete source code, will stimulate the generation of metabolism data and their release into the public domain.

## Data Availability

The source code of the approach presented in this work is available from https://github.com/molinfo-vienna/FAME.AL.

## Supporting Information Available

Performance progression of the active learning approach for splitting the active learning set and the validation set by atoms and by molecules; validation set performance as a function of the size of the active learning set used for training; 24 Sybyl atom types used by the CDPKit FAME descriptors; list of CDPKit 2D descriptors and their CDK counterparts; and performance of models as a function of the data sampling method and training set size (PDF)

## Dedication

We dedicate this work to the memory of Prof. Bernard Testa, who devoted much of his life to the research and teaching of xenobiotic metabolism, and who was a lead developer of the MetaQSAR database, which this and many other works build upon.
