# Supplementary material for: Active Learning Approach for Guiding Site-of-Metabolism Measurement and Annotation
Source: J Chem Inf Model. 2024 Jan 3;64(2):348–58. doi: 10.1021/acs.jcim.3c01588 (PMC10806800; doi:10.1021/acs.jcim.3c01588)
Supplement: Supplementary file 1 — ci3c01588_si_001.pdf [file ci3c01588_si_001.pdf]

Supporting information

Active learning approach for guiding site-of-metabolism measurement and annotation

Ya Chen,[1] Thomas Seidel,[1,2] Roxane Axel Jacob,[1,2,3] Steffen Hirte,[1,3] Angelica Mazzolari,[4] Alessandro Pedretti,[4] Giulio Vistoli,[4] Thierry Langer,[1,2] Filip Miljković,[5] Johannes Kirchmair[1,2*]

[1] Department of Pharmaceutical Sciences, Division of Pharmaceutical Chemistry, Faculty of Life Sciences, University of Vienna, Josef-Holaubek-Platz 2, 1090 Vienna, Austria
[2] Christian Doppler Laboratory for Molecular Informatics in the Biosciences, Department for Pharmaceutical Sciences, University of Vienna, 1090 Vienna, Austria
[3] Vienna Doctoral School of Pharmaceutical, Nutritional and Sport Sciences (PhaNuSpo), University of Vienna, 1090 Vienna, Austria
[4] Dipartimento di Scienze Farmaceutiche, Università degli Studi di Milano, I-20133 Milano, Italy
[5] Medicinal Chemistry, Research and Early Development, Cardiovascular, Renal and Metabolism (CVRM), BioPharmaceuticals R&D, AstraZeneca, Pepparedsleden 1, SE-43183 Gothenburg, Sweden

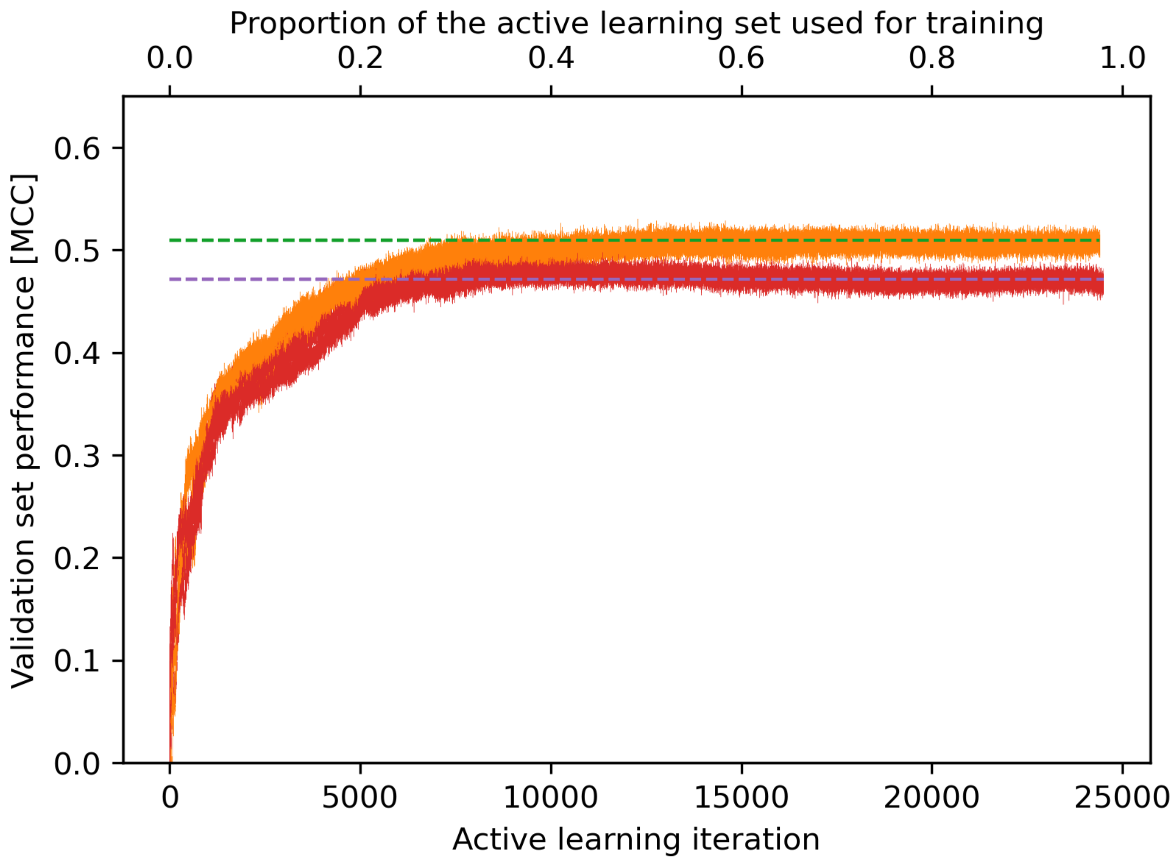

Figure S1. Performance progression of the active learning approach as different, randomly selected pairs of atoms are used as a starting point for active learning. The graph shows five repeats of the complete active learning process for splitting the active learning set and the validation set by atoms (orange) or by molecules (red), separately, as an example. The horizontal dashed line indicates the MCC obtained using the complete active learning set for model training.

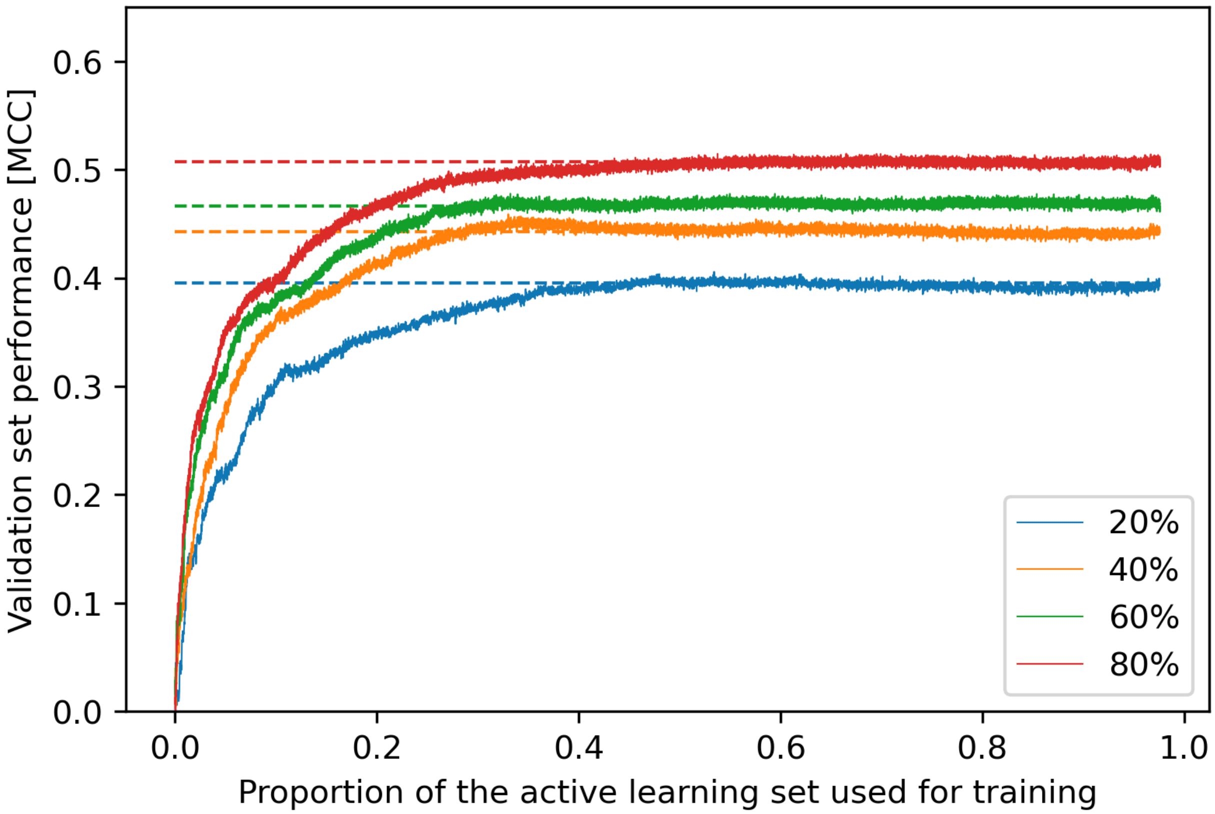

Figure S2. Validation set performance as a function of the size of the active learning set used for training. Reported are averages from five repeats with distinct initial atom pairs used as starting points for active learning. The horizontal dashed line indicates the MCC obtained using the complete active learning set for model training.

Table S1. The 24 Sybyl atom types used by the CDPKit FAME descriptors.

Index number in CDPKit	Sybyl atom type	Description
1	C.3	sp3 carbon
2	C.2	sp2 carbon
3	C.1	sp carbon
4	C.ar	aromatic carbon
6	N.3	sp3 nitrogen
7	N.2	sp2 nitrogen
8	N.1	sp nitrogen
9	N.ar	aromatic nitrogen
10	N.am	amide nitrogen
11	N.pl3	trigonal nitrogen
12	N.4	quaternary nitrogen
13	O.3	sp3 oxygen
14	O.2	sp2 oxygen
15	O.co2	carboxy oxygen
18	S.3	sp3 sulfur
19	S.2	sp2 sulfur
21	S.O2	sulfone sulfur
22	P.3	sp3 phosphorous
23	F	fluorine
38	Si	silicon
47	Cl	chlorine
48	Br	bromine
49	I	iodine
54	B	boron

Table S2. List of CDPKit 2D descriptors and their CDK counterparts.

(Atomic) Descriptor in CDPKit	Description	Corresponding descriptor in CDK (FAME 2 paper)
SybylType	Sybyl atom type, describing the element type and hybridization state	AtomType
HeavyAtomCount	Number of connected heavy atoms of an atom	AtomDegree
ExplicitValence	Formal valence of an atom	AtomValence
HybridPolarizability[1]	Hybridization state of an atom	atomHybridization
VSEPRCoordinationGeometry	VSEPR coordination geometry of an atom	atomHybridizationVSEPR
EffectivePolarizability[2]	Effective polarizability of a heavy atom	effectiveAtomPolarizability
-	-	stabilizationPlusCharge
InductiveEffect[3]	Inductive effect exerted on the atom	-
PEOESigmaCharge[4]	Sigma charge of a heavy atom	sigmaElectronegativity
PEOESigmaElectronegativity[4]	Sigma electronegativity of an atom	partialSigmaCharge
PiElectronegativity	Pi electronegativity of an atom	piElectronegativity
MMFF94Charge[5]	Partial charge of a heavy atom derived from the MMFF94	partialTChargeMMFF94
longestMaxTopDistInMolecule	Maximum number of bonds (topological distance) between two atoms in the molecule (i.e. the maximum in the topological distance matrix for the whole molecule)	longestMaxTopDistInMolecule
highestMaxTopDistInMatrixRow	Maximum number of bonds (topological distance) between two particular atoms in the molecule (maximum in the particular topological distance matrix row)	highestMaxTopDistInMatrixRow
diffSPAN	longestMaxTopDistInMolecule - highestMaxTopDistInMatrixRow	diffSPAN
relSPAN	highestMaxTopDistInMatrixRow / longestMaxTopDistInMolecule	relSPAN

Table S3. Performance of models as a function of the data sampling method and training set size (additional).

Data sampling method	% Data used for model training [a]	Positive label ratio for training data		Data set [b]	Predicted positive ratio [c]		Precision [c]	
		mean	std		mean	std	mean	std
Active learning	20/25	0.24	0.00	VS	0.11	0.00	0.56	0.00
Active learning	20/25	0.24	0.00	TS	0.11	0.00	0.55	0.01
Active learning	40/50	0.17	0.00	VS	0.11	0.01	0.57	0.02
Active learning	40/50	0.17	0.00	TS	0.11	0.00	0.56	0.01
Active learning	60/75	0.13	0.00	VS	0.11	0.01	0.57	0.01
Active learning	60/75	0.13	0.00	TS	0.11	0.00	0.55	0.01
Random selection	20/25	0.12	0.00	VS	0.10	0.01	0.51	0.02
Random selection	20/25	0.12	0.00	TS	0.10	0.00	0.49	0.02
Random selection	40/50	0.12	0.00	VS	0.10	0.01	0.54	0.02
Random selection	40/50	0.12	0.00	TS	0.10	0.01	0.53	0.01
Random selection	60/75	0.11	0.00	VS	0.11	0.01	0.56	0.02
Random selection	60/75	0.11	0.00	TS	0.11	0.00	0.54	0.02
n/a [d]	80/100	0.11	0.00	VS	0.11	0.01	0.57	0.01
n/a [d]	80/100	0.11	0.00	TS	0.11	0.00	0.54	0.01
n/a [e]	100/n/a [e]	0.12	n/a [e]	TS	0.11	n/a [e]	0.55	n/a [e]

[a] Of the training set for the baseline model/of the active learning set. For active learning, the training set used for generating the baseline model was further divided into an active learning set and a validation with a ratio of 80:20.
[b] VS: validation set; TS: test set.
[c] Performance averaged over 5 runs, each using a different fold as the validation set.
[d] The complete active learning data set is used for model training.
[e] The complete training set for the baseline model; hence, the results are the performance of the baseline model on the test set.

References

(1) Miller, K. J. Additivity Methods in Molecular Polarizability. *J. Am. Chem. Soc.* **1990**, 112, 8533–8542.

(2) PETRA Manual. https://www2.chemie.uni-erlangen.de/software/petra/manual/manual-16.html#P3538_49135 (accessed 2023-09-18).

(3) Hutchings, M. G.; Gasteiger, J. Residual Electronegativity - an Empirical Quantification of Polar Influences and Its Application to the Proton Affinity of Amines. *Tetrahedron Lett.* **1983**, 24, 2541–2544.

(4) Gasteiger, J.; Marsili, M. Iterative Partial Equalization of Orbital Electronegativity—a Rapid Access to Atomic Charges. *Tetrahedron* **1980**, 36, 3219-3228.

(5) Halgren, T. A. Merck Molecular Force Field. I. Basis, Form, Scope, Parameterization, and Performance of MMFF94. *J. Comput. Chem.* **1996**, 17, 490–519.
